# Ground Control System for UAS Safe Landing Area Determination (SLAD) in Urban Air Mobility Operations †

**DOI:** 10.3390/s22093226

**Published:** 2022-04-22

**Authors:** Gennaro Ariante, Salvatore Ponte, Umberto Papa, Alberto Greco, Giuseppe Del Core

**Affiliations:** 1 Department of Science and Technology, University of Naples “Parthenope”, 80133 Naples, Italy; umberto.papa@collaboratore.uniparthenope.it (U.P.); alberto.greco@uniparthenope.it (A.G.); giuseppe.delcore@uniparthenope.it (G.D.C.); 2 Department of Engineering, University of Campania “L. Vanvitelli”, 81031 Aversa, Italy; salvatore.ponte@unicampania.it

**Keywords:** UAV, UAS, LiDAR, urban air mobility, safe landing area determination, obstacle detection and avoidance

## Abstract

The use of the Unmanned Aerial Vehicles (UAV) and Unmanned Aircraft System (UAS) for civil, scientific, and military operations, is constantly increasing, particularly in environments very dangerous or impossible for human actions. Many tasks are currently carried out in metropolitan areas, such as urban traffic monitoring, pollution and land monitoring, security surveillance, delivery of small packages, etc. Estimation of features around the flight path and surveillance of crowded areas, where there is a high number of vehicles and/or obstacles, are of extreme importance for typical UAS missions. Ensuring safety and efficiency during air traffic operations in a metropolitan area is one of the conditions for Urban Air Mobility (UAM) operations. This paper focuses on the development of a ground control system capable of monitoring crowded areas or impervious sites, identifying the UAV position and a safety area for vertical landing or take-off maneuvers (VTOL), ensuring a high level of accuracy and robustness, even without using GNSS-derived navigation information, and with on-board terrain hazard detection and avoidance (DAA) capabilities, in particular during operations conducted in BVLOS (Beyond Visual Line Of Sight). The system is composed by a mechanically rotating real-time LiDAR (Light Detection and Ranging) sensor, linked to a Raspberry Pi 3 as SBC (Session Board Controller), and interfaced to a GCS (Ground Control Station) by wireless connection for data management and 3-D information transfer.

## 1. Introduction

The use of autonomous, semi-autonomous or remotely controlled Unmanned Aerial Vehicles (UAVs) has considerably increased during the last years, thanks to ease of use, flexibility and versatility, low price, and advances in battery endurance, motor, stabilization techniques, navigation, and onboard sensor technology [1]. UAV refers only to the vehicle, whereas the acronym UAS (Unmanned Aircraft System) considers the whole system, composed by the aircraft, a Ground Control Station (GCS), and a communication subsystem (data link) to send and receive information [2,3,4,5].

Small VTOL (Vertical Takeoff and Landing) aerial platforms are being used on a daily basis for several civil (professional, scientific, and recreational) and military applications, such as search and rescue missions, pipeline inspection, urban traffic monitoring, aerial mapping, surveillance of archaeological sites, control of the territory against environmental crimes, control of public order, and others. In the next decade, a further widespread diffusion of UAVs is expected [6]; on the other hand, their misuse can lead to perform anti-social, unsafe, and even criminal actions, such as privacy violation, collision hazard (with people, other UAVs, and manned aircrafts), and even transport of illicit materials and/or explosives or biological agents [7]. Therefore, it is mandatory to monitor these platforms in the airspace in the best way. Table 1 shows the most widespread classification of unmanned vehicles [8,9].

Currently, UASs are among the most used for civil applications, in individual missions or in swarms [10]. In the framework of a UAS autonomous flight mission in Urban Air Mobility operations, it is important to identify a safe area for a landing maneuver, to avoid potential conflicts with people, other vehicles, or structures [11,12,13,14]. Developing autonomous landing systems is the greatest challenge [15]; DAA (Detect and Avoid) or SAA (Sense and Avoid) systems, performing environment recognition during the landing phase by means of onboard sensors or by acquiring information from a ground station which could send safe paths to the vehicle, are of great importance and usefulness. Typically, environment perception is accomplished onboard the UAVs by means of real-time, small-dimension sensors (cameras, stereo cameras, Infra-Red (IR) or Time-of-Flight (ToF) cameras, ultrasound sensors, IR sensors, radar, LiDAR, etc.), easily configurable for UAV payloads [16,17,18,19]. These sensors could also be used to build small airfields equipped with GCA (Ground Controlled Approach)-like functionality. Nowadays, most of Safe Landing Area Determination (SLAD) systems are composed by technologies mounted on board. GNSS (Global Navigation Satellite System) technology is typically the primary source of positioning for most air and ground vehicles, and for a growing number of UASs in urban areas [20].

For optimal tracking and detection of landing areas, in urban operations, or during particular flight conditions (i.e., emergency cases, batteries with limited endurance, system failure), it is necessary to cover a large area in a short time. High maneuverability of small UAVs makes the tracking problem more difficult, due to the impossibility of making strong assumptions about the expected UAV motion trajectories and the related equations. Moreover, being pilotless and with no relevant payload, UAVs are aerial targets with small physical size, compared to conventional aircraft. Their identification and classification by High Range Resolution Radar Profiles (HRRPs) is problematic, and sub-centimeter resolution is required to capture spatial structures of targets with dimensions less than 100 cm.

In literature, a largely used approach to safe landing area identification involves vision-based systems and image analysis, and processing techniques [21,22,23,24,25], with alternative methodologies such as multi-sensor data fusion, using images and GNSS position data [26,27], point cloud reconstruction from LiDAR or radar sensors [28,29,30].

This paper exploits the ability of 2D LiDAR technology to provide three-dimensional elevation maps of a landing area and high-precision distance measurements, in order to design a safe landing identification strategy for UAS missions. A mechanically rotating, wide field-of-view 2D LiDAR is used, sensing the surroundings of the surveillance volume with high temporal resolution to detect obstacles, track objects, and support path planning. Another important issue, goal of this paper, is related to automatic landing zone surveying through obstacle detection around the landing area, in order to perform safe landing area determination (SLAD). Obstacle detection and avoidance systems (DAA) can be installed onboard the unmanned aircraft [31,32], and research on rotating LiDARs used onboard UAVs combined with stereo cameras for onboard landing area detection are available in literature [33,34]. In this paper, we propose a ground-controlled, rather than onboard-controlled, approach. The safe landing functionality, in terms of assessing the UAV correct position within a clear area, is performed by a LiDAR-equipped ground station, interfaced to a communication link with the UAV, therefore capable of identifying and transmitting safe landing paths to the approaching aircraft by identifying clearance areas, i.e., areas with no obstacles which could impair descent and/or landing. By mounting the LiDAR vertically on a servo motor, we combine the (now vertical) fast laser scanned information with the slow, controlled horizontal rotation of the motor, obtaining a tridimensional map of the surroundings of the landing zone. With respect to onboard SLAD systems, a ground-based solution offers many advantages, such as payload reduction, improved aircraft endurance due to reduced power consumption, communication of useful auxiliary information to the aircraft, and management of more than one aircraft in the surveillance volume. The system can identify obstacle-free areas, detect the UAS position, and indicate safe trajectories for landing maneuvers (a conceptual architecture of the system was presented by the authors in [35]).

This paper focuses on the system development, describing the theoretical and experimental phases towards the capability of creating a 3D point cloud and correcting for distortion and tilt, to obtain accurate estimates of the UAV distance to the ground and to the landing area, together with a preliminary obstacle detection strategy based on point cloud comparison. The innovative contributions of this research are:

- Promoting a new installation method for auxiliary equipment (a 2D rotating LiDAR with 3D capabilities, able to provide a point cloud of a volume in the vicinity of the landing site), aimed at improving the autonomous navigation capabilities of a VTOL small UAV (quadcopter, hexacopter, etc.), without installing additional sensors on the aircraft: therefore saving payload mass and power consumption, and enhancing the flight time (endurance).
- Proposing the application of safe landing trajectories as a function of the dynamic identification of obstacles in the surveillance volume (humans, hazards, etc.), both in indoor and outdoor scenarios, which is a significant improvement with respect to the ordinary vertical landing path, typical of other onboard autolanding systems. In principle, the LiDAR-equipped ground station could provide safe landing information, even for missions where the UAV is supposed to land on a moving platform. This work presents the design aspects and a validation of the ground system, in terms of providing a point cloud of the 3D surveillance volume (including the landing site), detecting the UAV as it enters the landing volume, estimating its position with respect to the landing site, identifying objects which could impair the safe landing, and monitoring the descent of the flying robot.
- Proposing a ground system capable of providing a safe descent path even in the presence of onboard hardware/software errors (for example, actuator failure, loss of position estimation from a possible IMU embedded in the UAV flight control system, etc.), by providing timely autonomous identification of safe paths, and assisting a controlled descent and land on the ground. Our approach is inspired by typical landing systems for civil aviation, such as ILS (Instrument Landing System) or GCA (Ground Controlled Approach).
- Proposing a simple and cost-effective alternative to vision-based SLAD methods.
- Developing a simple, cheap and useful obstacle detection system by analyzing the point cloud characteristics obtained by the ground-based LiDAR, with a clustering algorithm based on difference “images” of two corrected point cloud scenarios, with and without the obstacle.
- Promoting a methodology of safe landing area determination in GPS-denied environments, in failure scenarios, or when the aircraft is not equipped with a precise localization system, therefore extending the application domain of UAVs/UASs, especially multirotor helicopters.
- Proposing a potential minimum landing time approach for small, fast-moving small/micro UAVs in a variety of environments (typical flight speed of 10 m/s and 30 min battery life).

**Figure 1:**
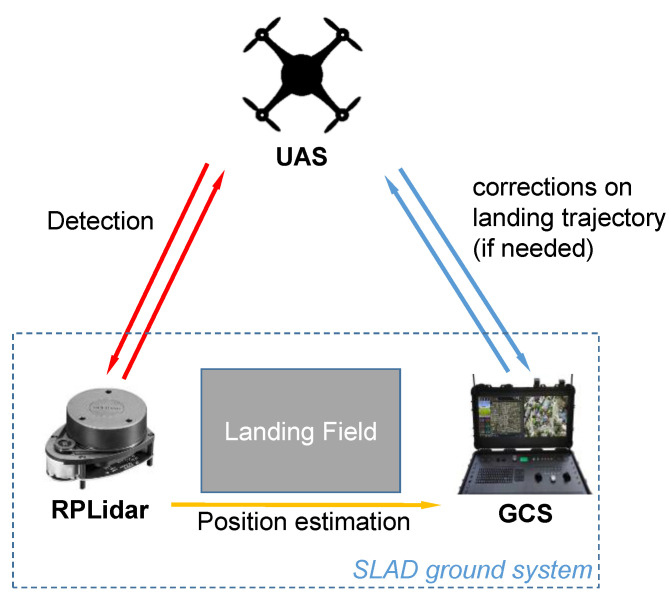
Architecture of the SLAD system.

The architecture of the ground-based safe landing assessment methodology is shown in Figure 1. The Ground Control Station (GCS) establishes a data link with the UAV, to send and receive information.

The paper is organized as follows. Section 2 quickly recaps the theoretical framework and the observation geometry. In Section 3, the hardware (electronic components) and methods used for the preliminary setup of our LiDAR-based ground station are discussed in detail. Section 4 presents the point correction procedure and the obstacle detection algorithm implemented, together with experimental results from data collection campaigns which validate the feasibility of the proposed methodologies. Further developments and concluding remarks are outlined in Section 5.

## 2. Theoretical Framework

**Figure 2:**
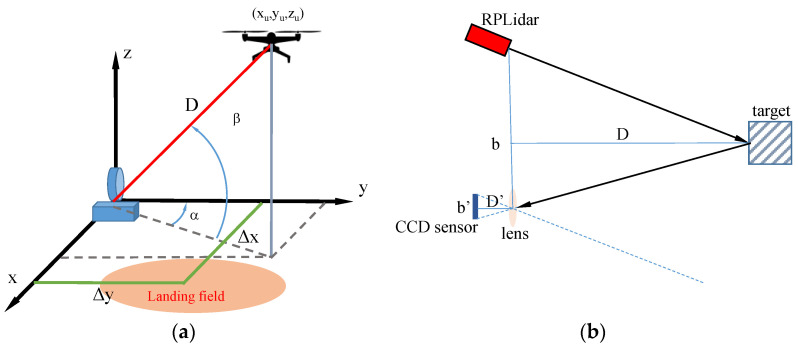
(**a**) UAV position estimation by LiDAR measurements; (**b**) laser triangulation principle.

Figure 2a depicts the observation geometry. The LiDAR is in the origin of the reference frame, the UAV position is given by ($x_{u},\;y_{u},\;z_{u}$), α is the servo motor angle, *β* and the slant range D are estimated by the laser sensor, rotating around an axis parallel to the *xy*-plane, and the co-ordinates of the center L of the landing area are (Δx, Δy). For the sake of simplicity, the landing field and the LiDAR sensor are supposed coplanar, but the generalization for Δz≠0 (i.e., a difference in height between the landing field and the sensor) is straightforward.

Denoting with $\bar{UL}$ the slant range (or LOS, Line Of Sight) of the UAV from the landing center, and with $\bar{LR}$ the ground range, we have:

(1) $$\{\begin{array}{c} x_{u}=D\cos\beta \sin\alpha \\ y_{u}=D\cos\beta \cos\alpha \\ z_{u}=\frac{D}{\sin\beta }\\ \bar{UL}=\sqrt{{\left(x_{u}-\Delta x\right)}^{2}+{\left(y_{u}-\Delta y\right)}^{2}+z_{u}^{2}}\\ \bar{RL}=\sqrt{{\left(x_{u}-\Delta x\right)}^{2}+{\left(y_{u}-\Delta y\right)}^{2}} \end{array}$$

Figure 2b shows the principle of laser triangulation, a typical alternative to ToF (Time-of-Flight) measurement in low-cost laser sensors. A lens with a focal distance *d’*, placed at a distance *b* from the laser source, focalizes the returned ray on a CCD (CMOS)-based position-sensitive device (PSD) at distance *d’* from the lens. The triangles defined by (*b*, *d*) and (*b’*, *d’*) are similar, and the distance to the object is nonlinearly proportional to the angle of the reflected light (i.e., the laser acceptance angle). The perpendicular distance to the object (*d*) is given by:

(2) $$d=\frac{b\;d’}{b’}$$

if *β* is the angle of the laser beam, the slant range (distance along the geometric ray) is:

(3) $$D=\frac{d}{\sin\beta }$$

From (2) and (3), the range sensitivity (or resolution) Δd/Δb’ is equal to:

(4) $$\frac{\Delta d}{\Delta b’}=-\frac{d^{2}}{bd’}$$

and grows quadratically with the distance from the object. Small values of bd’ allow measurement of small distances, whereas the sensor resolution is enhanced by large bd’.

## 3. SLAD Ground System

The SLAD ground system is composed by:

− RPLiDAR sensor, model A1M8;
− Raspberry Pi 3 (SBC);
− 5-A Power Module;
− Servo motor with standing structure;
− PC-based Ground Control Station to manage LiDAR data and send clearance data (safe paths, safe landing zone) to the UAV;
− Communication subsystem for real-time data transfer from the sensor to the GCS and transmission of safety information to the UAV.

### 3.1. LiDAR Sensor: RPLIDAR Model A1M8

**Figure 3:**
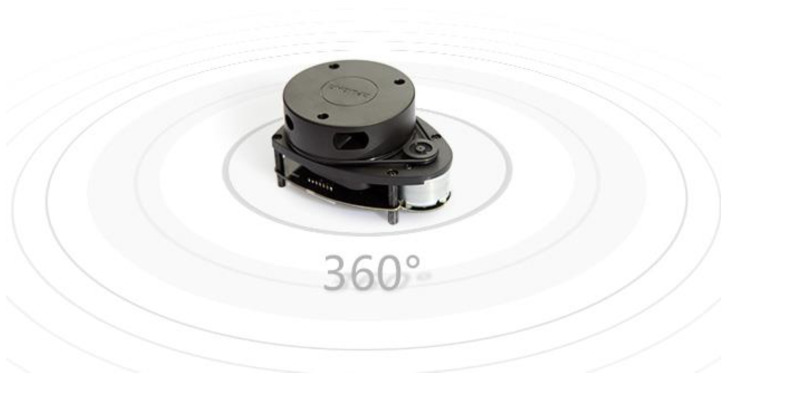
RPLIDAR A1M8 (2D rotating laser scanner).

The low-cost, 360-degree FOV (Field Of View) laser range scanner RPLIDAR Model A1M8, built by Shanghai Slamtec Co., Shanghai, China, [36], shown in Figure 3, has been widely used in a variety of applications in robotics, in particular in the framework of SLAM (Simultaneous Localization and Mapping) methodologies for mobile robots [37]. It measures the distance from an object emitting a laser beam which is reflected by the object surface and measured and received by a position-sensitive detector. Built-in circuitry calculates the distance from the object by means of triangulation, based on the position of the detector with respect to the reflected light, obtaining range and angular data in the sensor coordinate system.

The active ranging device uses a low power (<5 mW peak) infrared laser (785-nanometer wavelength) as its light source, transmitting 110 µs pulses, and can perform 360-degree 2D scan within a 5 m range with angular resolution ≤1° [36,38]. Typical distance resolution is <0.5 mm, or less than 1% of the distance. The produced 2D point cloud data can be used in mapping, localization, and object/environment modeling. It is fully suitable as a fundamental part of the SLAD system developed in this paper.

**Figure 4:**
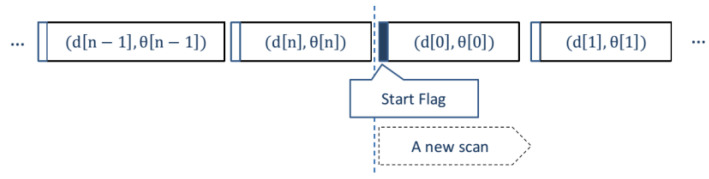
Data frames from the RPLIDAR A1M8.

RPLIDAR A1′s scan rate is 5.5 Hz (configurable up to 10 Hz) when sampling 360 points per scan, with a typical sample frequency of 4 kHz (1 sample every 250 µs). With 5 V supplied by the power module during our tests, the sensor rotates at 468 rpm (i.e., the scan frequency is 7.8 Hz, or 128 ms per revolution), collecting (on average) 515 samples per revolution (i.e., the sample frequency is approximately 4 kHz). The assembly contains a range scanner system and a motor with speed detection and adaptive system. It can operate excellently in both indoor and outdoor environments with no sunlight. The system automatically adjusts the laser scan rate according to the motor speed and uses UART serial port (115,200 bps rate) as the communication interface with host computers or controllers. After power-on, the sensor starts rotating and scanning clockwise. The distance data acquired can be stored on a PC or a microcontroller (e.g., Raspberry, Arduino) through the communication interface (USB connection). Table 2 reports information of each sample point, whereas Figure 4 visualizes the formatted dataflow.

**Figure 5:**
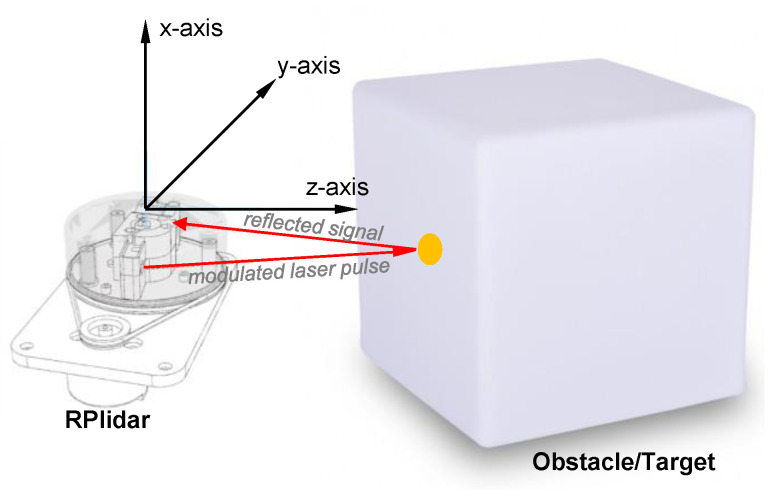
Laser triangulation principle.

The RPLIDAR A1 operates by measuring the angle of the reflected light (Figure 5), using high-speed vision acquisition and processing hardware, and outputting distance value and angle between the illuminated object and the sensor, thanks to a built-in angular encoding system. The whole system measures distance data more than 2000 times per second and high-resolution distance output (<1% of the distance). A 2 min pre-heating in start scan mode, with the sensor rotating, is recommended to obtain optimal measurement accuracy.

### 3.2. Raspberry PI 3

The Raspberry Pi 3 microcomputer hosts a 64-bit quad-core processor running at 1.4 GHz, dual-band 2.4 GHz, and 5 GHz wireless LAN, Bluetooth 4.2/BLE, faster Ethernet, and PoE (Power over Ethernet) capability via a separate PoE “hat”. The dual-band wireless LAN comes with modular compliance certification, allowing the board to be designed into end products with significantly reduced wireless LAN compliance testing, improving both cost and time to market [39]. The Raspberry Pi 3 is used in this paper as the main controller and transmitter of the data acquired by the RPLIDAR, and sent via its Wi-Fi module to a PC-based GCS for storage and processing. Raspberry and RPLIDAR are powered by a 5 VDC source through an embedded power module. The whole system is autonomous and portable without the constraint of a fixed power supply.

### 3.3. Power Module

**Figure 6:**
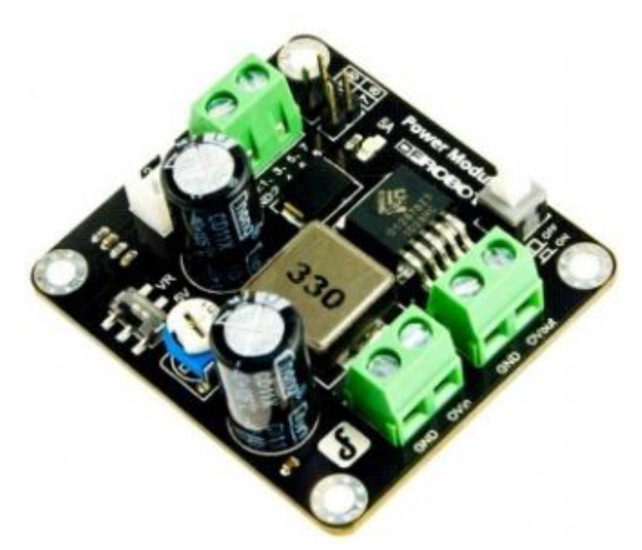
DFRobot 5-A Power Module DFR0205.

The DFR0205 Power Module (Figure 6), built by DFRobot, is based on a small size 350 kHz switching frequency PWM buck DC-to-DC Converter (GS2678 [40]). It can convert any DC voltage between 3.6 V and 25 V to a selectable voltage from 3.3 V to 25 V.

It is possible to choose 5 V direct output voltage with a switch or adjust the output voltage by means of a control resistor. The OV_out_ (Original Voltage output) interface can output the original voltage of input, so that it can be used as a power source for other modules. In our research, the DRF0205 was used to convert the 7.4 V input from a 2600-mAh Li-ion 18,650 battery (each battery is 3.7 V) to the 5 V output which supplies energy to the Raspberry Pi 3 and the servo motor. Laboratory tests showed that the system can work continuously for about 6 h.

### 3.4. Standing SLAD Structure

**Figure 7:**
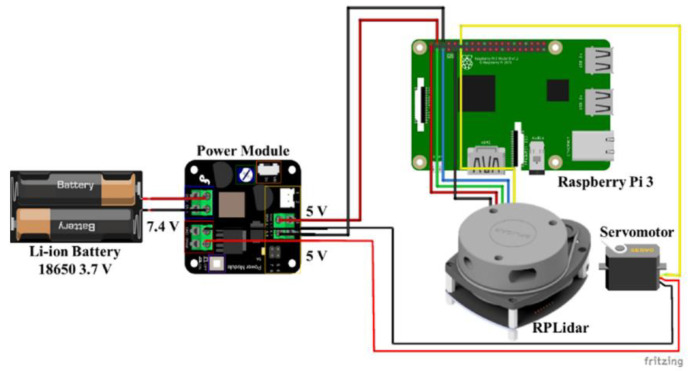
Electric connections of the SLAD system.

**Figure 8:**
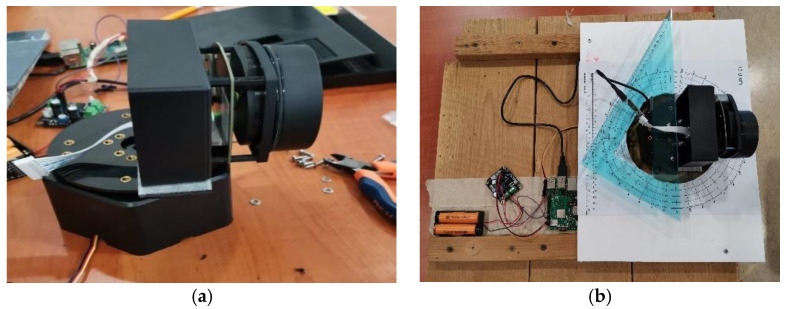
(**a**) Servo motor with rotating disc, to allow LiDAR rotation in the plane orthogonal to the laser rotation axis; (**b**) The prototype with a graduated disc allowing to take readings of the servo motor angular position α.

The electric scheme of the SLAD system (Raspberry, RPLIDAR, battery, servo motor and Power Module) is shown in Figure 7. The components were mounted on a rotating structure (a disc with a series of self-lubricating bearings to reduce friction and vibrations during rotation) locked to a servo motor. The whole system is mounted on a standing structure made in ABS (Figure 8).

**Figure 9:**
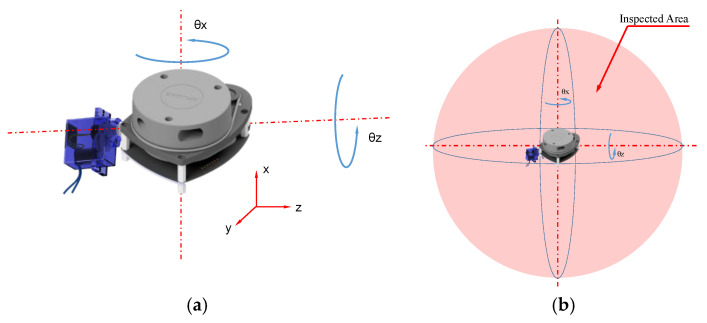
(**a**) Functionality of the system; (**b**) Survey volume.

Figure 9 shows the capability of acquiring tridimensional information by combining the rotation of the LiDAR and the servo motor, and the inspection volume of the system.

### 3.5. Communication Subsystem

**Figure 10:**
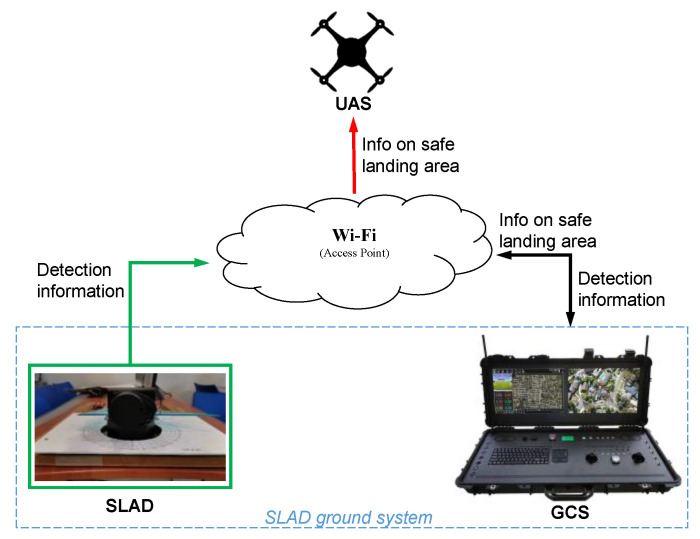
Block scheme of the communication subsystem.

The communication subsystem, depicted in Figure 10, is based on a common Wi-Fi network, which interconnects the SLAD system (managed by the Raspberry Pi 3) to a remote terminal (a PC-based GCS). After configuring Raspbian (the Raspberry operating system, stored in a SD card) with the SSID and password details of the local Wi-Fi and enabling SSH (the Secure Shell protocol, providing a secure channel with a cryptographic network protocol [41]), Raspberry Pi 3 will connect automatically to the Wi-Fi network after booting. With a simple Python interface, developed by the authors, Raspberry acquires real-time data from the RPLiDAR and sends them to the remote terminal, connected to the common Wi-Fi. Data acquisition is performed at 8 kHz sampling rate. Figure 10 shows a conceptual architecture of the communication subsystem.

## 4. Experimental Tests and Results

Simulation tests of the SLAD ground system have been performed at the Flight Dynamics Laboratory of the University of Naples “Parthenope”, Italy.

### 4.1. Validation of Laboratory Tests

**Figure 11:**
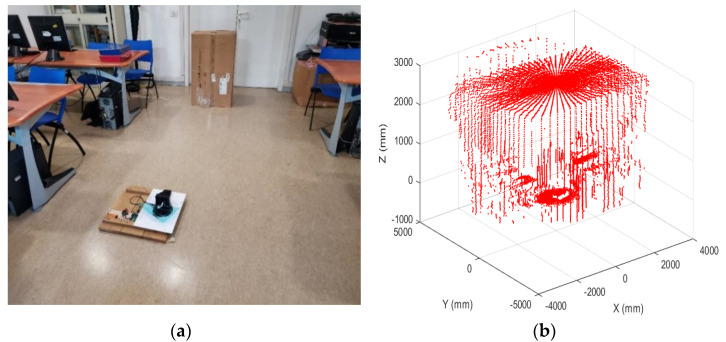
(**a**) Indoor experiment scenario (room dimensions: 6400 × 2890 mm); (**b**) 3D reconstruction from LiDAR data.

By using laboratory open spaces, a simulated airfield has been built up (Figure 11) to test the LiDAR scanning capability in an area surrounding an assigned landing field. Every data acquisition campaign had a duration of 1 min, and data were sampled at 4 kHz. The control volume was mapped by the system, and some obstacles with known dimensions were placed to verify the obstacle detection capability of the ground station. Reference distance measurement were collected with a laser distance meter (Bosch PLR 40C [42]). Post-processed data allowed us to reconstruct the 3D flight volume and evaluate possible nonlinear distortion effects, imprecise pointing accuracies, and distance measurement errors.

**Figure 12:**
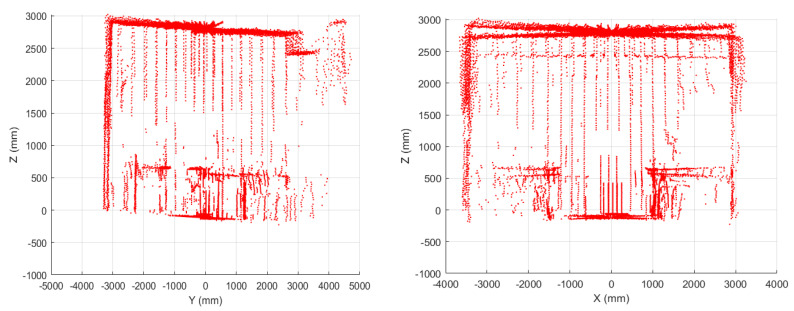
2D reconstructions from LiDAR data: planes yz and xz.

From data acquired in the yz- and xz-planes (Figure 12), tilt errors, and distortion effects were noted, pushing towards finding a calibration strategy, described in the next section.

### 4.2. SLAD System Extrinsic Calibration: Removal of Disalignment and Nonlinear Distortions

To compensate for tilt problems, related to suboptimal level of the mechanical structure, and to sensor position and installation issues, we devised a simple extrinsic calibration procedure, determining the transformation from the sensor measurements, i.e., the 3D coordinates of a point in the “image” derived from the laser data, to the 3D coordinates of the point in the sensor coordinate system. The calibration algorithm applies a translation ***t*** and a rotation ***R***:

(5) $${\left[x_{SLAD}\;\;y_{SLAD}\;\;z_{SLAD}\right]}^{T}=t_{SLAD}^{LDR}+R{\left[x_{LDR}\;\;y_{LDR}\;\;z_{LDR}\right]}^{T}$$

**Figure 13:**
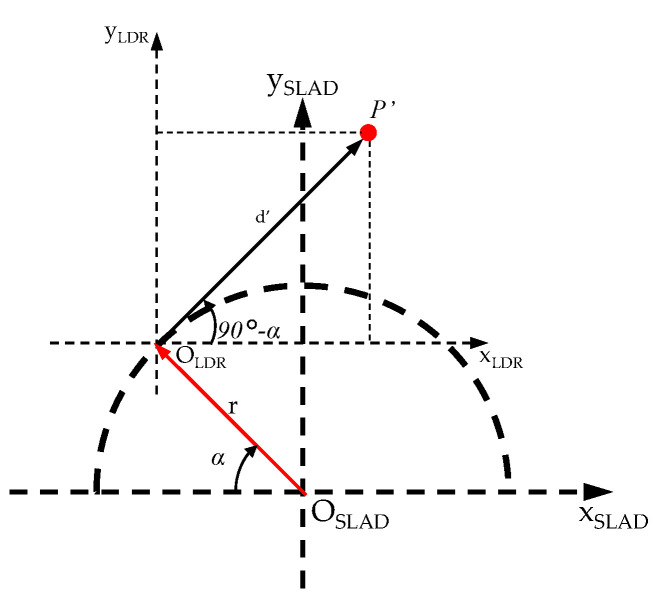
Coordinate frames.

The rotation angles (Φ, Θ, Ψ) to be estimated are derived from a linear regression analysis in the three orthogonal planes. The angular coefficients of the regression lines are evaluated with respect to the three axes $(x_{LDR},\;y_{LDR},\;z_{LDR}$). As far as the translation is concerned, indicating with *r* the distance between the origin of the SLAD reference and the LiDAR reference (equal to 85 mm), we have (Figure 13):

(6) $$x_{SLAD}=x_{LDR}+r*\cos\left(\alpha \right)$$

(7) $$y_{SLAD}=y_{LDR}+r*\sin\left(\alpha \right)$$

**Figure 14:**
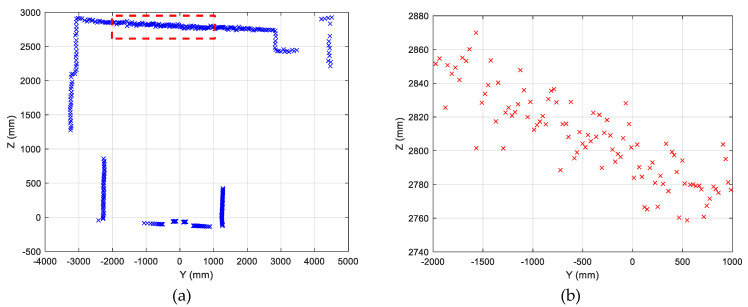
(**a**) Reference data used for the evaluation of the regression line in the yz-plane; (**b**) red dotted box zoom.

To evaluate the regression line, we chose, as a reference measurement, data representing distance from horizontal and vertical objects in the “real” world, i.e., walls and ceiling, as in Figure 14a (red dotted box).

**Figure 15:**
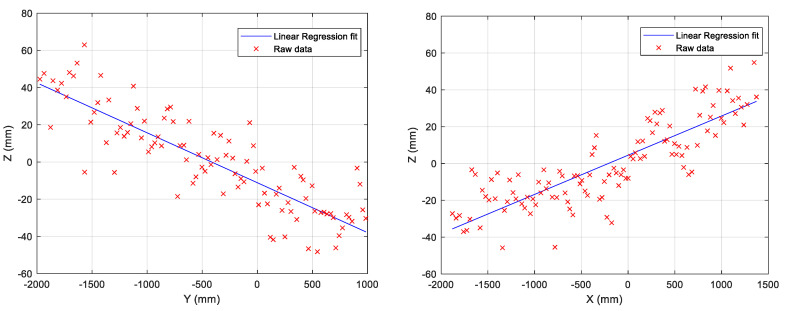
Regression lines for yz- and xz-planes.

The angular coefficient *m* and the constant term *q* of a generic straight line y=mx+q are found by minimizing the function:

(8) $$ℱ\left(m,q\right)=\overset{n}{\underset{i=0}{\sum }}\left(m*x_{i}+q-y_{i}\right){\;}^{2}$$

and are given by:

(9) $$m=\frac{\left(n+1\right)\sum_{i=0}^{n}x_{i}y_{i}-\sum_{i=0}^{n}x_{i}\sum_{i=0}^{n}y_{i}}{\left(n+1\right)\sum_{i=0}^{n}x_{i}^{2}-{\left(\sum_{i=0}^{n}x_{i}\right)}^{2}}$$

(10) $$q=\frac{\sum_{i=0}^{n}y_{i}\sum_{i=0}^{n}x_{i}^{2}-\sum_{i=0}^{n}x_{i}\sum_{i=0}^{n}x_{i}y_{i}}{\left(n+1\right)\sum_{i=0}^{n}x_{i}^{2}-{\left(\sum_{i=0}^{n}x_{i}\right)}^{2}}$$

where $\left(x_{i,}y_{i}\right)$ are the coordinates of a point in the “image” and *n*+1 is the number of measurements. Figure 15 shows the regression lines for xy- and yz-planes, Table 3 gives the numerical values of *m* and *p*, and Table 4 shows the corresponding angles of the regression lines.

**Figure 16:**
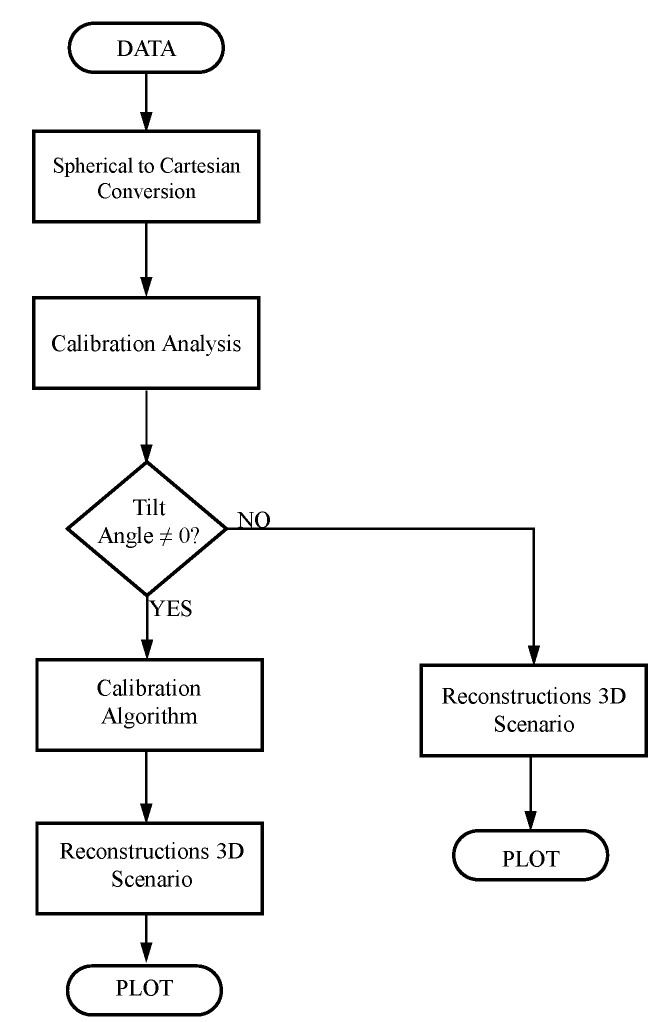
Flowchart of the extrinsic calibration procedure.

**Figure 17:**
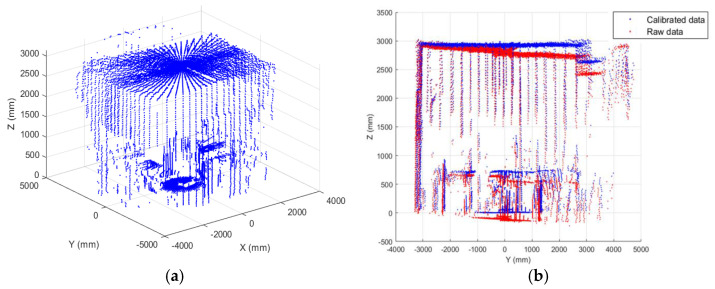
(**a**) 3D reconstruction scenario from calibrated data; (**b**) Comparison between raw and calibrated data (yz-plane).

**Figure 18:**
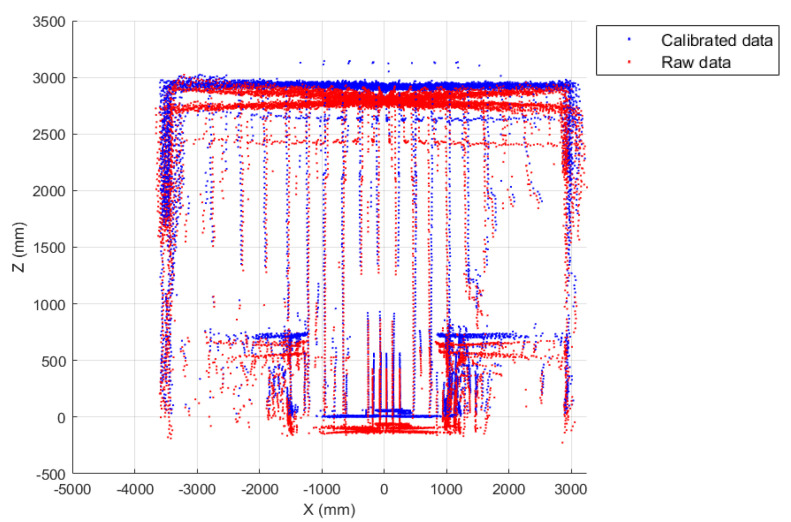
Comparison between raw and calibrated data (xz-plane).

The rotation matrices:

(11) $$R_{x}=\left[\begin{array}{cc} \begin{array}{c} 1\\ 0 \end{array} & \begin{array}{c} \begin{array}{cc} 0 & 0 \end{array}\\ \begin{array}{cc} \cos\Phi & -\sin\Phi \; \end{array} \end{array}\\ 0 & \;\begin{array}{cc} \;\sin\Phi & \;\cos\Phi \end{array} \end{array}\right]$$

(12) $$R_{y}=\left[\begin{array}{cc} \begin{array}{c} \cos\Theta \\ 0 \end{array} & \begin{array}{c} \begin{array}{cc} 0 & \;\sin\Theta \end{array}\\ \begin{array}{cc} 1 & 0 \end{array} \end{array}\\ -\sin\Theta & \begin{array}{cc} 0 & \cos\Theta \end{array} \end{array}\right]$$

are applied to every point in the data measurement space. Figure 16 depicts a flowchart of the calibration procedure, whereas Figure 17 and Figure 18 show the results of the point correction procedure.

### 4.3. Obstacle Detection Methodology

We devised a simple and utilizable obstacle detection procedure that provides effective obstacle information for the approaching UAV, based on the difference between two scenarios scanned by the LiDAR and stored in point clouds (without obstacles and with obstacles in the landing area). With respect to other techniques (for example, IMU/INS data associated with the laser scanning [43], multi-point cloud fusion [44], clustering algorithms [45], and convolutional networks [46]), which are CPU-time consuming and unfit to UASs with low computing capabilities, we followed an approach based on a simple range difference between neighboring point in scan angle [47]. The technique, developed in the MATLAB environment, is based on a co-registration between the two point clouds by using linear interpolation of the single LiDAR scan for each angle value of the servo motor.

**Figure 19:**
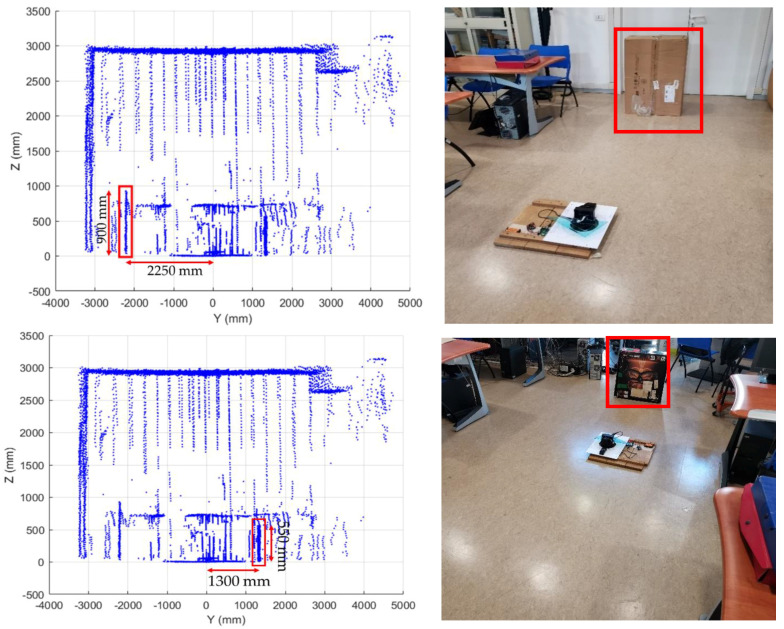
LiDAR mapping of the simulated landing field with two obstacles.

**Figure 20:**
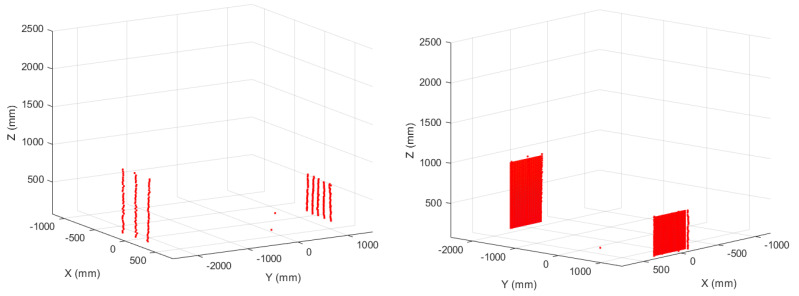
Sample points relative to the two obstacles.

We placed two obstacles in the landing scenario, with dimensions 900 × 600 mm at a distance of 2250 mm from the SLAD system, and 550 × 550 mm at a distance of 1300 mm. Figure 19 (red boxes) shows the obstacle positions in the corrected point clouds, and Figure 20 shows the result of the “difference image” between the clean landing field and the same field with the obstacles.

### 4.4. Outdoor Validation Test

**Figure 21:**
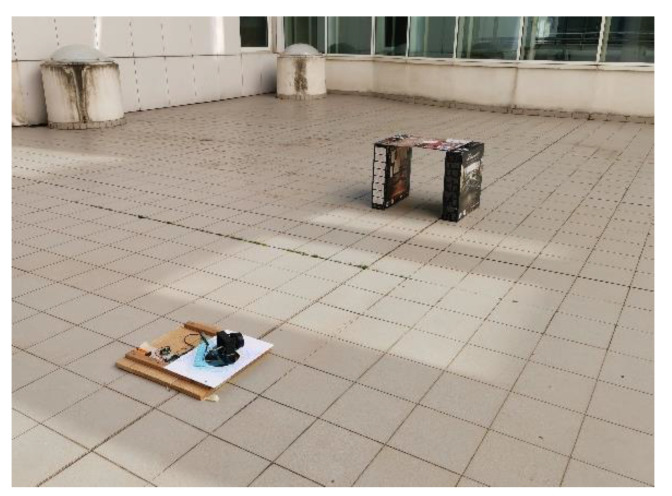
LiDAR calibration during the outdoor tests.

In order to identify landing vehicles and map their descent trajectory, we set up a simple outdoor test by mapping a surveillance volume with the system, with the UAV approaching the landing field and executing a vertical landing. A preliminary calibration of the LiDAR was performed to verify the correctness of the alignment and the absence of geometric distortions. A sample object was used to test the calibration procedure (Figure 21). The calibration procedure described in the previous section gave the values shown in Table 5 or the inclination angles.

**Figure 22:**
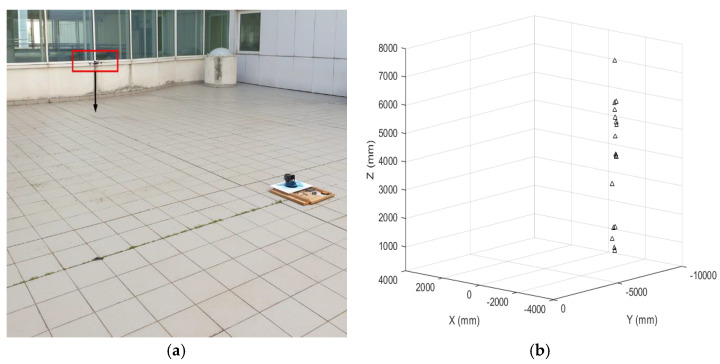
(**a**) UAV landing path; (**b**) Reconstruction from LiDAR data.

After extrinsic calibration, a UAV entered the scenario (highlighted in the red box in Figure 22a), and data were acquired from the ground system when the aircraft reached a distance from the ground of 7000 mm. Post-processed data are shown in Figure 22b.

## 5. Conclusions and Further Work

This work presents the design and validation of a simple and cost-effective 2D LiDAR-based ground system for Safe Landing Area Determination for small UAVs, capable of providing a point cloud of the 3D surveillance volume reconstructed from 2D laser distance measurements by triangulation, using a servo motor to rotate the sensor (a low-cost RPLIDAR A1M8, developed by Slamtec Co., Shanghai, China) along the vertical axis. The system can detect the UAV as it enters the landing volume, to estimate its position with respect to the landing site, and to monitor the descent of the flying robot. Data collection was managed by a Raspberry Pi 3 microcomputer. A point cloud calibration procedure allowed correct reconstruction of the 3D scenario, by reducing tilt errors of the mechanical structure and geometric distortions. An obstacle detection strategy based on simple clustering by means of differences between homologous points in a reference point cloud, and a scenario with obstacles, demonstrates the capability of the system in terms of detecting and signaling obstacles to the approaching aircraft. The application of safe landing trajectories as a function of the dynamic identification of obstacles (humans, hazards, etc.) in a surveillance volume surrounding the landing site, both in indoor and outdoor scenarios, is a significant improvement from the ordinary vertical landing path, typical of other autolanding systems onboard the aircraft. In principle, the LiDAR-equipped ground station could provide safe landing information, even for missions where the UAV is supposed to land on a moving platform. Sending corrections to the landing trajectory to the approaching UAV via a communication link is the main aspect to be implemented in the successive stages of the research.

Ground-based safe landing area determination has the advantage of reducing the payload onboard the aircraft, which only needs a communication link to exchange information with the station located in the vicinity of the landing field. Outdoor tests verified the capability of the system to track the approaching vehicle, and derive information on the landing path and the distance from the landing field.

The detection of obstacles on the ground and/or in the surveillance volume is only a preliminary test to evaluate the system capability to de-risk the potential challenges involved in the UAV approach and landing procedure. The main objective remains to assist and give guidance to a flying robot in the terminal flight phase. Obstacle detection plays a fundamental role in the conception of the ground system, which will provide clearance signals to the approaching vehicle and transmit information about a possible safe descent.

Further developments will involve refinements of the mechanical structure (with a precision encoder for better knowledge of the angular position of the servo motor, for example) and enhancement of the calibration/reconstruction strategy, by means of filtering techniques to reduce measurement noise and possible effects of radial distortions in the LiDAR lens.

## Figures and Tables

**Table 1:** NATO Classification of UAS (AGL = Above Ground Level; BLOS = Beyond Line Of Sight).

Class	Category	Normal Employment	Normal Operating Altitude	Normal Operating Radius	Primary Supported Commander	Example Platform
III (>600 kg)	Strike/combat	Strategic/National	up to 65,000 ft	Unlimited (BLOS)	Theatre	Reaper
	HALE (High Altitude, Long Endurance)	Strategic/National	up to 65,000 ft	Unlimited (BLOS)	Theatre	Global Hawk
	MALE (Medium Altitude, Long Endurance)	Operational/Theatre	up to 45,000 ft	Unlimited (BLOS)	JTF	Heron
II (150 kg–600 kg)	Tactical	Tactical Formation	up to 18,000 ft AGL	200 km (LOS)	Brigade	Hermes 450
I (<150 kg)	Small (>15 kg)	Tactical Unit	up to 5000 ft AGL	50 km (LOS)	Battalion, Regiment	Scan Eagle
	Mini (<15 kg)	Tactical Subunit (manual or hand launch)	up to 3000 ft AGL	Up to 25 km (LOS)	Company, Platoon, Squad	Skylark
	Micro ^1^ (<66 J)	Tactical Subunit (manual or hand launch)	up to 200 ft AGL	Up to 5 km (LOS)	Platoon, Squad	Black Widow

^1^ UAS that have a maximum energy state less than 66 J are not likely to cause significant damage to life or property, and do not need to be classified or regulated for airworthiness, training, etc.

**Table 2:** The RPLIDAR A1 Sample point data information.

Data Type	Unit	Description
Distance, d	mm	Current measured distance value
Heading, θ	degrees	Current heading angle of the measurement
Quality	level	Quality of the measurement
Start Flag	(Boolean)	Flag of a new scan

**Table 3:** Parameters of the regression lines in the xz- and yz-planes.

Axis	m	q
Y-Z	−0.021	2796
X-Z	0.020	2799

**Table 4:** Tilt angles of the regression lines.

Angle	Value (deg)
Φ	−1.23
Θ	1.15

**Table 5:** Inclination angles of the regression line in the outdoor calibration test.

Angle	Value (deg)
Φ	1.02
Θ	1.08
